# *Panax notoginseng saponins* mitigate cisplatin induced nephrotoxicity by inducing mitophagy via HIF-1α

**DOI:** 10.18632/oncotarget.19900

**Published:** 2017-08-03

**Authors:** Xueyan Liang, Yufang Yang, Zhenguang Huang, Jinling Zhou, Yue’e Li, Xiaobin Zhong

**Affiliations:** ^1^ Department of Pharmacy, The First Affiliated Hospital of Guangxi Medical University, Nanning, China; ^2^ Postgraduate, Department of Pharmacy, The First Affiliated Hospital of Guangxi Medical University, Nanning, China; ^3^ Regenerative Medicine Research Center of Guangxi Medical University, Nanning, China

**Keywords:** panax notoginseng saponins, cisplatin-induced nephrotoxicity, mitophagy, HIF-1α

## Abstract

We investigated the role of HIF-1α in the mitigation of cisplatin-induced nephrotoxicity by *Panax notoginseng saponins* (PNS) in a rat model. Serum creatinine (Scr), blood urea nitrogen (BUN) and urinary N-acetyl-β-D-glucosaminidase (NAG) levels were all elevated in cisplatin treated rats. PNS reduced Scr, BUN and NAG levels in the presence or absence of the HIF-1α inhibitor 2-methoxyestradiol (2ME2). PNS also reduced the high tubular injury scores, which corresponded to renal tubular damage in cisplatin-treated rats and which were exacerbated by 2ME2. Renal tissues from PNS-treated rats showed increased HIF-1α mRNA and nuclear localized HIF-1α protein. Moreover, PNS treatment increased BNIP3 mRNA as well as LC3-II, BNIP3 and Beclin-1 proteins and the LC3-II/LC3-I ratio in rat renal tissues. This suggested that PNS treatment enhanced HIF-1α, which in turn increased autophagy. This was confirmed in transmission electron micrographs of renal tissues that showed autophagosomes in PNS-treated renal tissues. These findings demonstrate that PNS mitigates cisplatin-induced nephrotoxicity by enhancing mitophagy via a HIF-1α/BNIP3/Beclin-1 signaling pathway.

## INTRODUCTION

Cisplatin (cis-diamminedichloroplatinum II) is a commonly used chemotherapeutic drug for solid tumors [1]. However, the renal toxicity of cisplatin limits its clinical application [2]. The precise mechanism of cisplatin-induced nephrotoxicity (CIN) has not been fully understood. Previous studies showed the involvement of oxidative stress [3], inflammation [4] and apoptosis [5] as plausible mechanisms of CIN. Moreover, effective therapeutic strategies are not available as yet for CIN. Therefore, novel therapeutic agents are needed for treating CIN in patients that need cisplatin-based chemotherapy. Recent studies have shown that autophagy protects against cisplatin-induced acute kidney injury [6]. Further, mitophagy, which removes damaged mitochondria also protects against CIN [7].

*Panax notoginseng saponins* (PNS) are active ingredients extracted from *Panax notoginseng*, which has been extensively used for hundreds of years in China to treat coronary heart disease, hemorrhagic disorders and ischemic cerebrovascular diseases [8]. Recent studies have shown that PNS attenuates cancer growth [9], protects against CIN and enhances the antitumor effects of cisplatin [10, 11]. In our previous study, we postulated that PNS alleviated CIN by enhancing hypoxia inducible factor-1α (HIF-1α)-mediated mitophagy [12]. Therefore, in this study we investigated the role of HIF-1α in mediating CIN protection by PNS in a rat model.

## RESULTS

### PNS reduces Scr, BUN and urinary NAG levels in cisplatin treated rats

**Figure 1:**
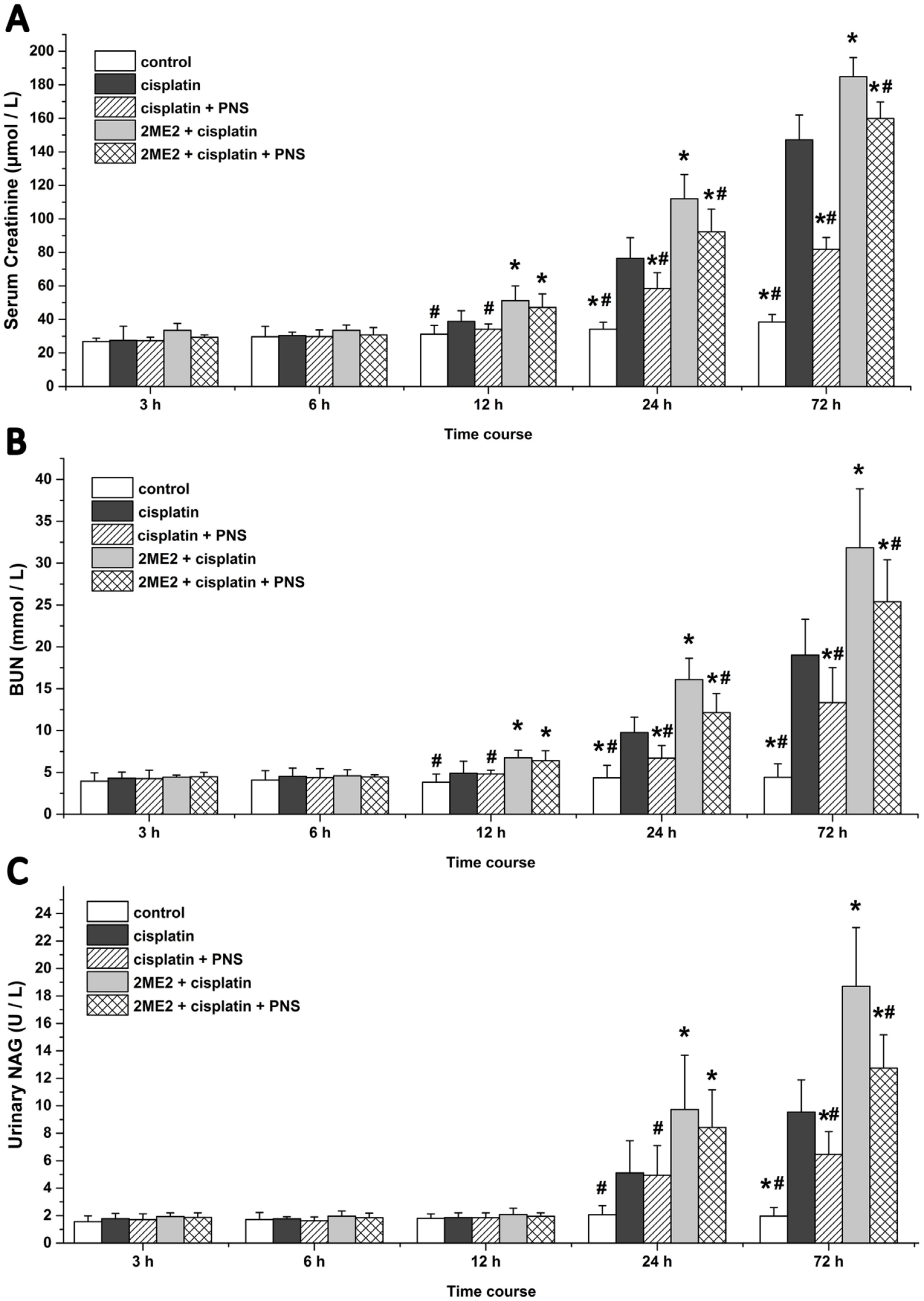
Serum creatinine, BUN and urinary NAG levels in rats at different time points **(A)** Scr **(B)** BUN and **(C)** urinary NAG levels in control, cisplatin, cisplatin + PNS, 2ME2 + cisplatin and 2ME2 + cisplatin + PNS groups of rats are shown. Note: Data are represented as mean ± SD (n = 6). ^*^ denotes *P* < 0.05 compared with the cisplatin group, ^**#**^ denotes *P* < 0.05 compared with the 2ME2 + cisplatin group.

The cisplatin group rats showed higher serum creatinine (Scr) and blood urea nitrogen (BUN) levels at 24h and 72h in addition to increased N-acetyl-β-D-Glucosaminidase (NAG) levels at 72h compared to the control group (Figure 1). Compared with the cisplatin group, the 2ME2 + cisplatin group rats showed further elevated Scr and BUN levels from 12h to 72h in addition to increased NAG at 24h and 72h (Figure 1). However, the cisplatin + PNS group demonstrated lower Scr and BUN levels at 24h and 72h as well as reduced NAG at 72h compared to the cisplatin group. The 2ME2 + cisplatin + PNS group also demonstrated lower Scr and BUN levels at 24h and 72h as well as reduced NAG at 72h compared to the 2ME2 + cisplatin group (Figure 1).

### PNS reduces cisplatin induced renal tissue damage

**Figure 2:**
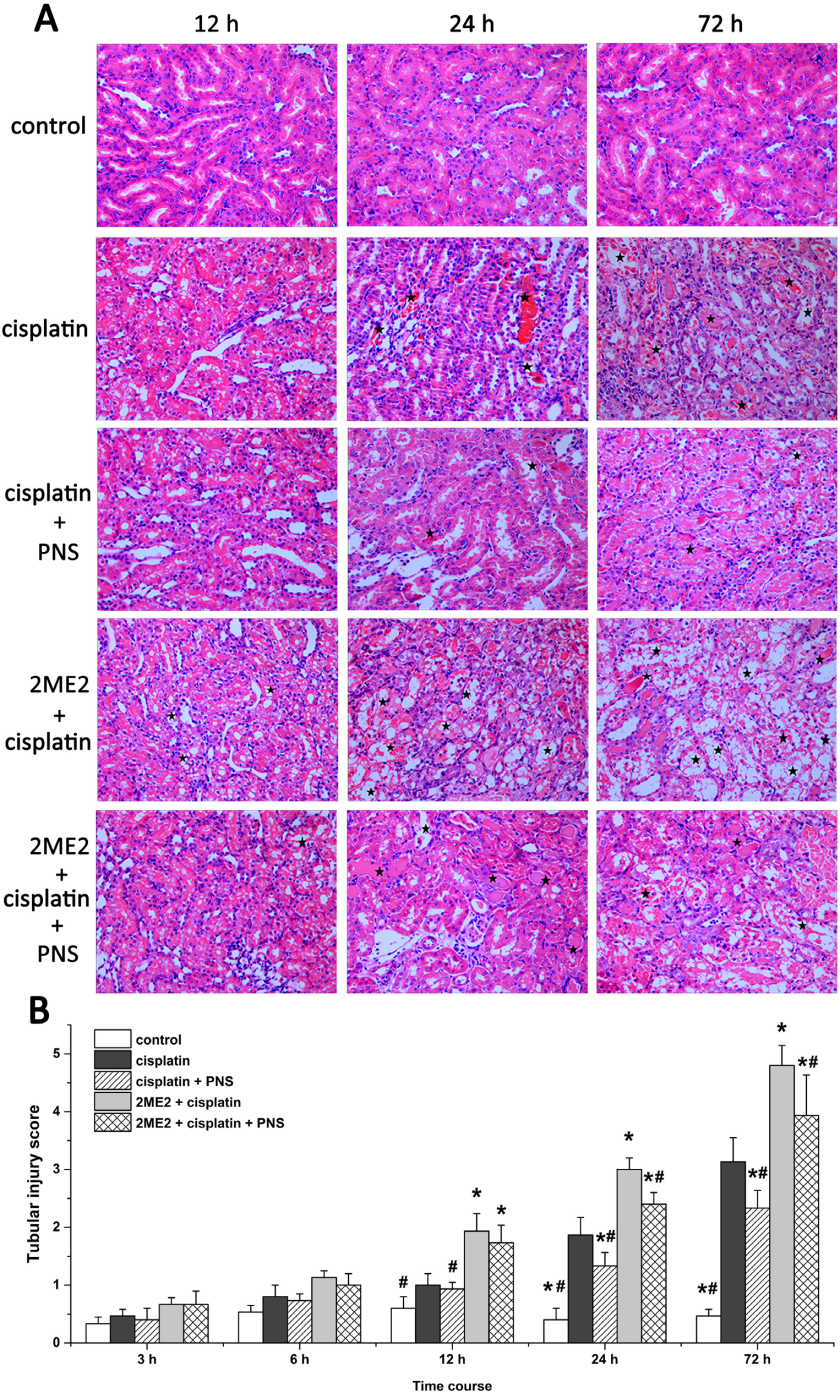
Histological analysis of H&E stained renal tissue samples and their tubular injury scores **(A)** Representative images (400×) of H&E stained renal tissue samples from control, cisplatin, cisplatin + PNS, 2ME2 + cisplatin and 2ME2 + cisplatin + PNS groups of rats at different time points. The stars indicate the injured regions. **(B)** Tubular injury scores in control, cisplatin, cisplatin + PNS, 2ME2 + cisplatin and 2ME2 + cisplatin + PNS groups of rats at different time points. Note: Data are represented as mean ± SD (n = 6).^*^ denotes *P* < 0.05 compared with the cisplatin group; ^**#**^ denotes *P* < 0.05 compared with the 2ME2+ cisplatin group.

Analysis of hematoxylin and eosin (H&E) stained renal tissue samples showed that cisplatin treatment resulted in protein or red blood cell casts with some renal tubular epithelial cells showing signs of edema, detachment, degeneration or necrosis from 12h to 72h compared to controls (Figure 2A). The renal injury was more pronounced in the 2ME2 + cisplatin group compared to the cisplatin group from 12h to 72h. Conversely, at 24h and 72h, the cisplatin + PNS group exhibited less renal injury than the cisplatin group, and the 2ME2 + cisplatin + PNS group exhibited less renal injury than the 2ME2 + cisplatin group (Figure 2A).

Higher tubular injury scores were observed in the cisplatin group at 24h and 72h compared to the control group. Moreover, the 2ME2 + cisplatin group showed higher injury scores than the cisplatin group from 12h to 72h. Conversely, the cisplatin + PNS group had lower injury scores at 24h and 72h compared to the cisplatin group. The 2ME2 + cisplatin + PNS group also had lower injury scores at 24h and 72h compared to the 2ME2 + cisplatin group (Figure 2B).

### PNS increases autophagy in cisplatin treated renal tubular epithelial cells

**Figure 3:**
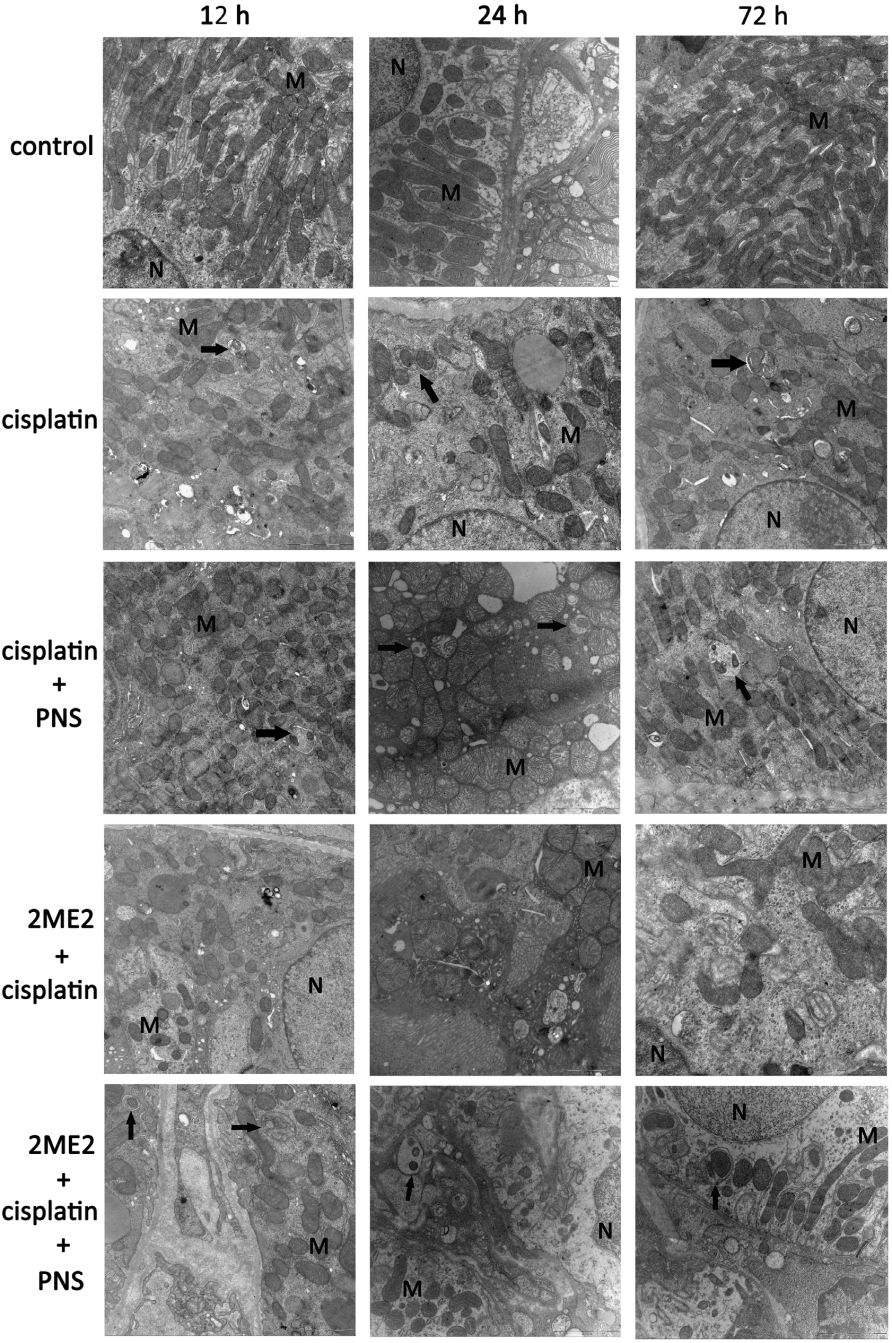
Transmission electron micrographs of rat renal tissue sections Representative TEM images (20000×) of renal tissue sections from control, cisplatin, cisplatin + PNS, 2ME2 + cisplatin and 2ME2 + cisplatin + PNS groups of rats at different time points are shown (scale:1 μm). Note: Black arrows denote autophagosomes; N denotes nucleus; M denotes mitochondria.

Transmission electron micrograph (TEM) images revealed swollen mitochondria with vague, fractured or absent cristae in the cisplatin group compared to the control (Figure 3). The 2ME2 + cisplatin group showed more damaged mitochondria compared to the cisplatin group from 12h to 72h. Conversely, the cisplatin + PNS group showed reduced mitochondrial damage compared to the cisplatin group. The 2ME2 + cisplatin + PNS group also showed reduced mitochondrial damage compared to the 2ME2 + cisplatin group (Figure 3).

TEM images also showed autophagosomes in the cisplatin, cisplatin + PNS and 2ME2 + cisplatin + PNS groups, whereas autophagosome was not found in the control and 2ME2 + cisplatin groups (Figure 3).

### PNS increases LC3-II expression and LC3-II/LC3-I ratio in cisplatin treated renal tissues

**Figure 4:**
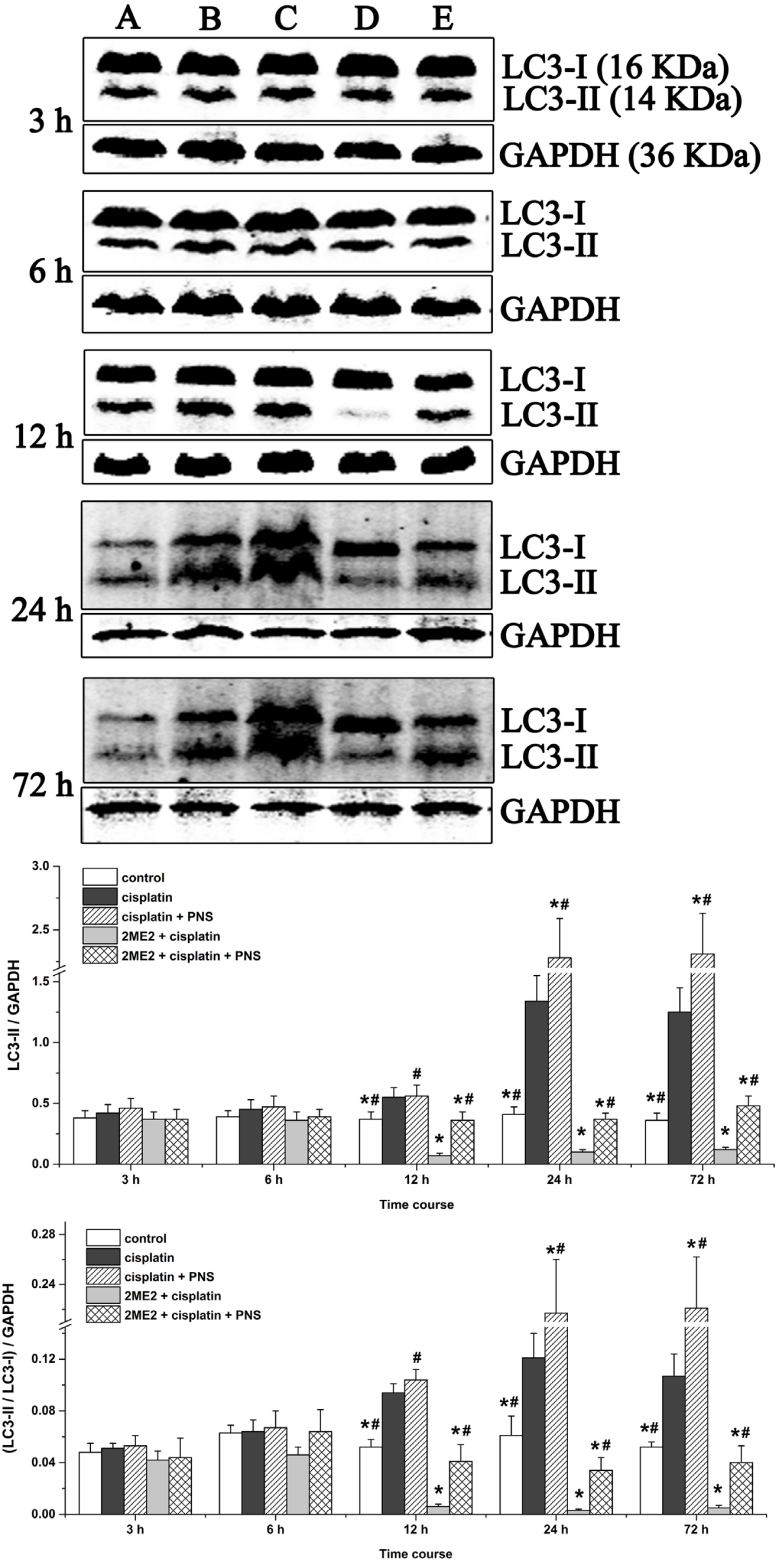
Western blotting analysis of LC3 expression in rat renal tissues LC3 expression in renal tissues from (**A)** control, (**B)** cisplatin, (**C)** cisplatin + PNS, (**D)** 2ME2 + cisplatin, (**E)** 2ME2 + cisplatin + PNS groups is shown. Note: Data are represented as mean ± SD (n = 4). ^*^ denotes *P* < 0.05 compared with the cisplatin group; ^**#**^ denotes *P* < 0.05 compared with the 2ME2 + cisplatin group.

To gain insights on the status of autophagy, we analyzed microtubule-associated protein-1 light chain 3 (LC3-I and LC3-II) by western blotting. We observed increased LC3-II levels and LC3-II/LC3-I ratio in renal tissues from the cisplatin group (12h to 72h) compared to the control (Figure 4). However, the 2ME2 + cisplatin group showed decreased LC3-II expression and LC3-II/LC3-I ratio compared to the cisplatin group (Figure 4). Conversely, we observed increased LC3-II expression and the LC3-II/LC3-I ratio in the cisplatin + PNS group (24h and 72h) compared to the cisplatin group, as well as in the 2ME2 + cisplatin + PNS group (12h to 72h) compared to the 2ME2 + cisplatin group (Figure 4).

### PNS increases HIF-1α mRNA levels in cisplatin treated renal tissues

**Figure 5:**
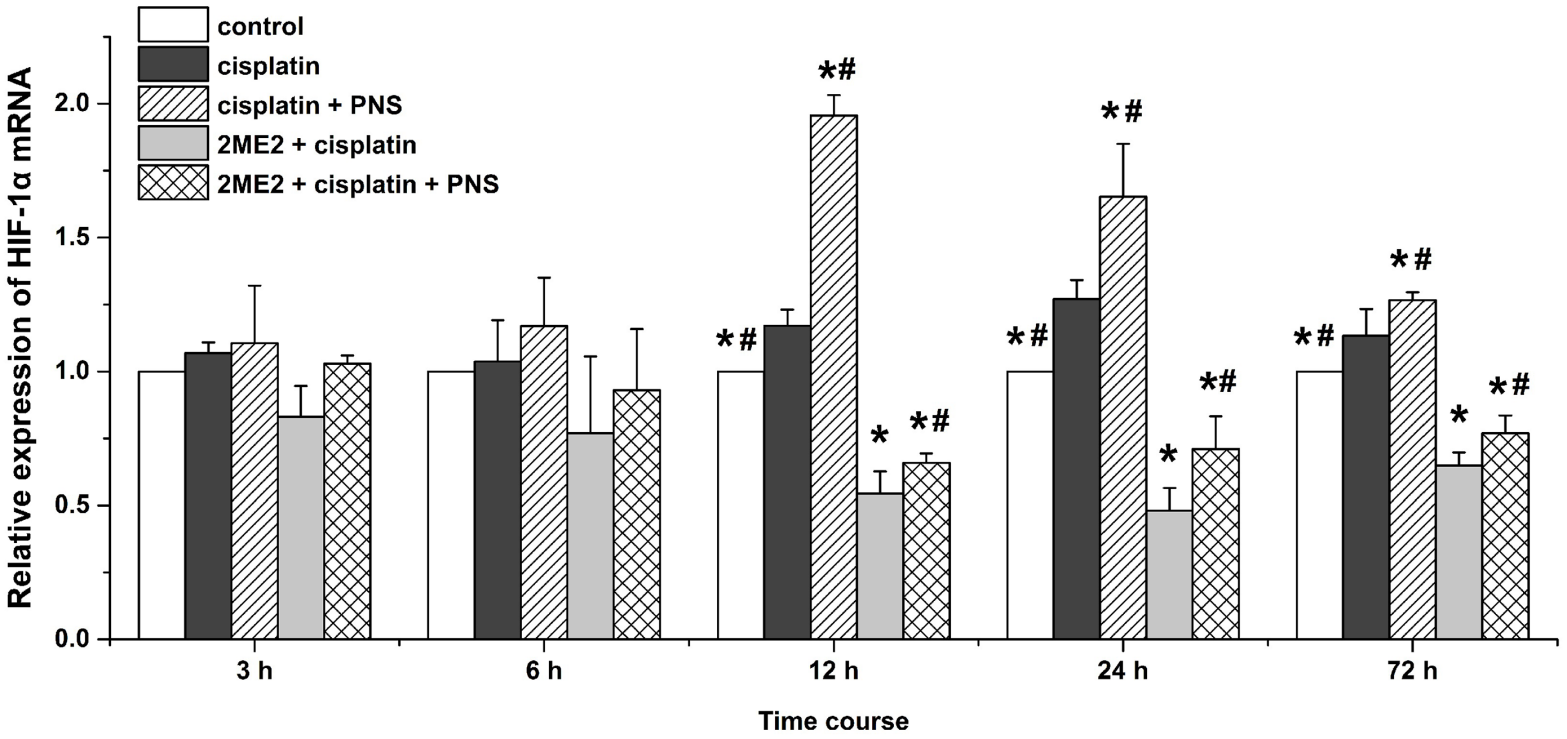
Quantitative RT-PCR analysis of HIF-1α mRNA expression Relative expression of HIF-1α mRNA in renal tissues from control, cisplatin, cisplatin + PNS, 2ME2 + cisplatin and 2ME2 + cisplatin + PNS groups of rats at different time points is shown. Note: Data are represented as mean ± SD (n = 3). ^*^ Denotes *P* < 0.05 when compared with the cisplatin group, ^#^ denotes *P* < 0.05 when compared with the 2ME2 + cisplatin group.

Next, we detected HIF-1α mRNA levels by quantitative real-time polymerase chain reaction (qRT-PCR) in the various rat groups (Figure 5). We observed increased HIF-1α mRNA expression in the cisplatin group compared to the control group from 12h to 72h (Figure 5). HIF-1α mRNA levels were decreased in the 2ME2 + cisplatin group compared to the cisplatin group from 12h to 72h (Figure 5). Moreover, higher HIF-1α mRNA levels were observed in the cisplatin + PNS group (12h to 72h) compared to the cisplatin group, as well as in the 2ME2 + cisplatin + PNS group (12h to 72h) compared to the 2ME2 + cisplatin group (Figure 5).

### PNS increases nuclear localization of HIF-1α in cisplatin treated renal tissues

**Figure 6:**
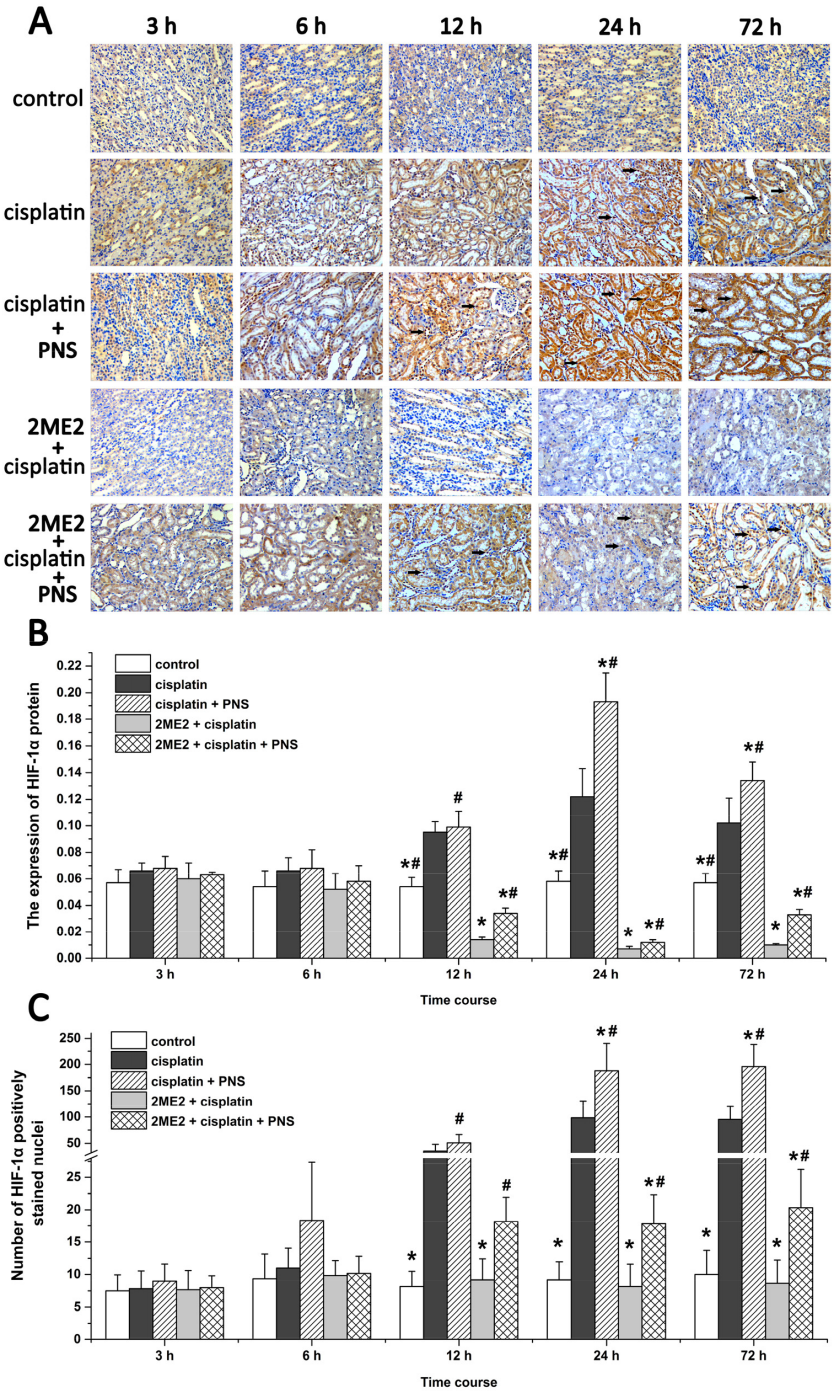
Immunohistochemical analysis of HIF-1α in rat renal tissues **(A)** Representative images (400×) of immunohistochemically stained renal tissue sections from control, cisplatin, cisplatin + PNS, 2ME2 + cisplatin and 2ME2 + cisplatin + PNS groups of rats. **(B)** The expression of HIF-1α protein and **(C)** nuclear localization of HIF-1α in renal tissues from control, cisplatin, cisplatin + PNS, 2ME2 + cisplatin and 2ME2 + cisplatin + PNS groups of rats are shown. Note: Black arrows denote nuclear HIF-1α staining. Data are represented as mean ± SD (n = 6). ^*^ denotes *P* < 0.05 compared with the cisplatin group; ^#^ denotes *P* < 0.05 when compared with the 2ME2 + cisplatin group.

Next, we analyzed expression and nuclear localization of HIF-1α by immunohistochemistry. The HIF-1α protein was mainly expressed in renal tubular epithelial cells (Figure 6A). The cisplatin group showed increased HIF-1α expression compared to the control group (Figure 6A and 6B). However, its expression was decreased in the 2ME2 + cisplatin group compared to the cisplatin group (Figure 6A and 6B). HIF-1α levels were higher in the cisplatin + PNS group (24h and 72h) compared to the cisplatin group, as well as in the 2ME2 + cisplatin + PNS group (12h to 72h) compared to the 2ME2 + cisplatin group (Figure 6A and 6B).

In the renal tissues from the control group, HIF-1α protein was localized predominantly in the cytoplasm (Figure 6A). The cisplatin group (12h to 72h) showed increased nuclear localization of HIF-1α compared to the control group (Figure 6A and 6C). However, the 2ME2 + cisplatin group (12h to 72h) showed decreased nuclear localization of HIF-1α compared to the cisplatin group. Moreover, we observed increased nuclear HIF-1α in the cisplatin + PNS (24h and 72h) compared to the cisplatin group, as well as in the 2ME2 + cisplatin + PNS group (12h to 72 h) compared to the 2ME2 + cisplatin group (Figure 6A and 6C).

### PNS increases BNIP3 mRNA and protein levels in cisplatin treated rat renal tissues

**Figure 7:**
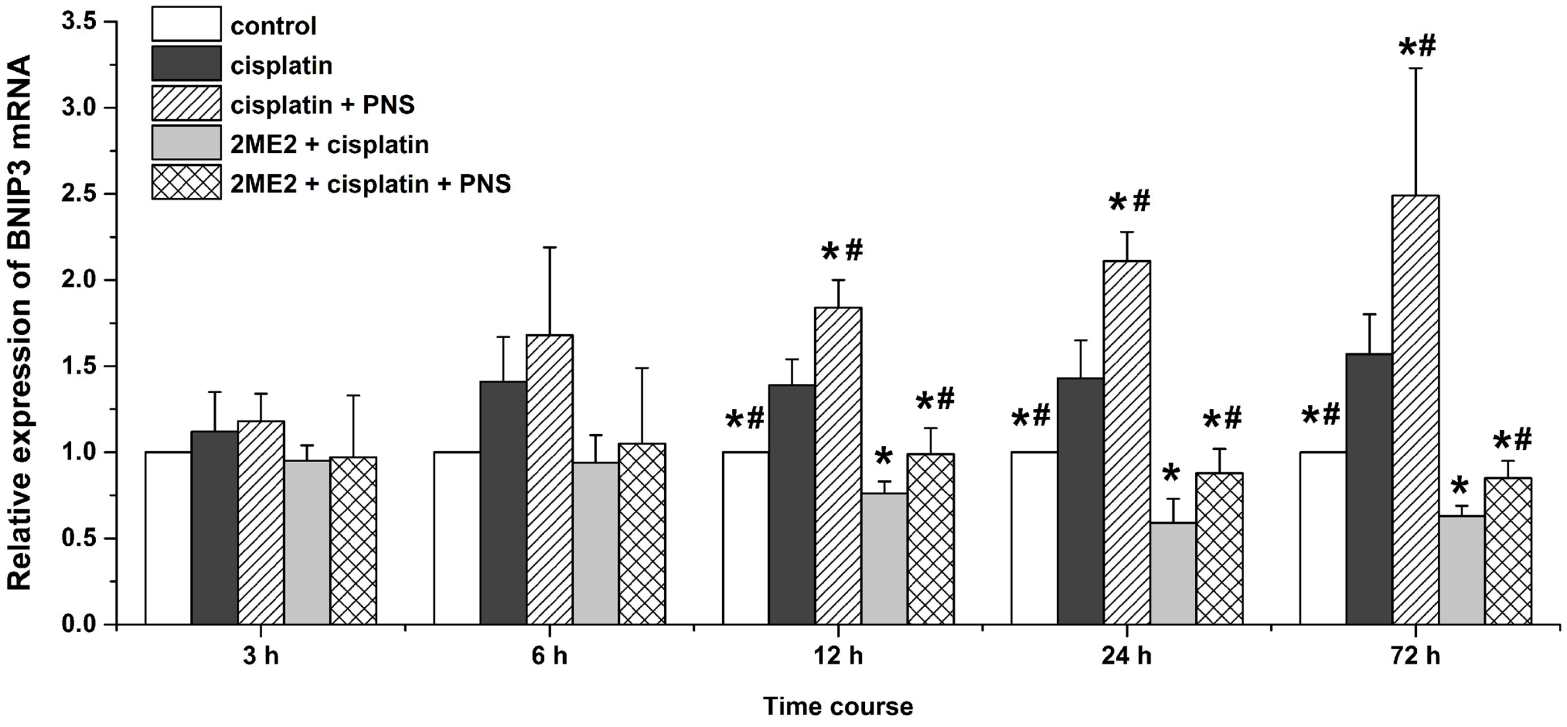
Quantitative RT-PCR analysis of BNIP3 mRNA expression in rat renal tissue samples Relative expression of BNIP3 mRNA in renal tissues from control, cisplatin, cisplatin + PNS, 2ME2 + cisplatin and 2ME2 + cisplatin + PNS groups of rats at different time points is shown. Note: Data are represented as mean ± SD (n = 3). ^*^ denotes *P* < 0.05 compared with the cisplatin group; ^#^ denotes *P* < 0.05 when compared with the 2ME2 + cisplatin group.

**Figure 8:**
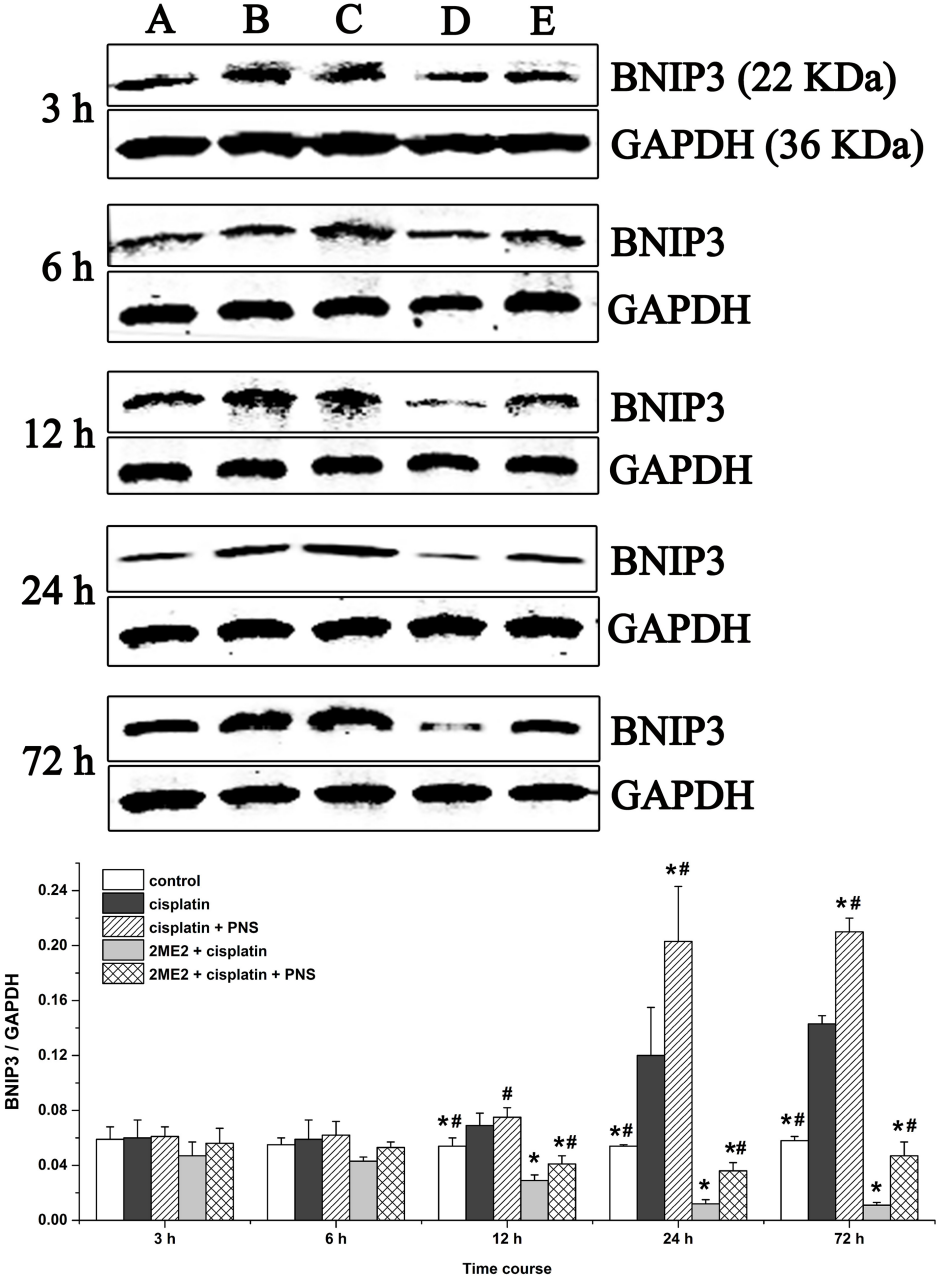
Western blotting analysis of BNIP3 expression in rat renal tissues Quantitative analysis of BNIP3 expression in renal tissues from (**A)** control, (**B)** cisplatin, (**C)** cisplatin + PNS, (**D)** 2ME2 + cisplatin, (**E)** 2ME2 + cisplatin + PNS groups is shown. Note: Data are represented as mean ± SD (n = 4). ^*^ denotes *P* < 0.05 compared with the cisplatin group; ^#^ denotes *P* < 0.05 when compared with the 2ME2 + cisplatin group.

Next, we analyzed expression of BCL2/adenovirus E1B 19kDa-interacting protein 3 (BNIP3) mRNA (Figure 7) and protein (Figure 8) levels in renal tissues from the different rat groups by qRT-PCR and western blotting, respectively. We observed increased BNIP3 mRNA and protein levels in the cisplatin group compared to the control (Figures 7 and 8). On the other hand, BNIP3 mRNA and protein levels were decreased in the 2ME2 + cisplatin group compared to the cisplatin group (Figures 7 and 8). Further, BNIP3 mRNA and protein levels were higher in the cisplatin + PNS compared to the cisplatin group, as well as in the 2ME2 + cisplatin + PNS group compared to the 2ME2 + cisplatin group (Figures 7 and 8).

### PNS increases Beclin-1 levels in cisplatin treated rat renal tissues

**Figure 9:**
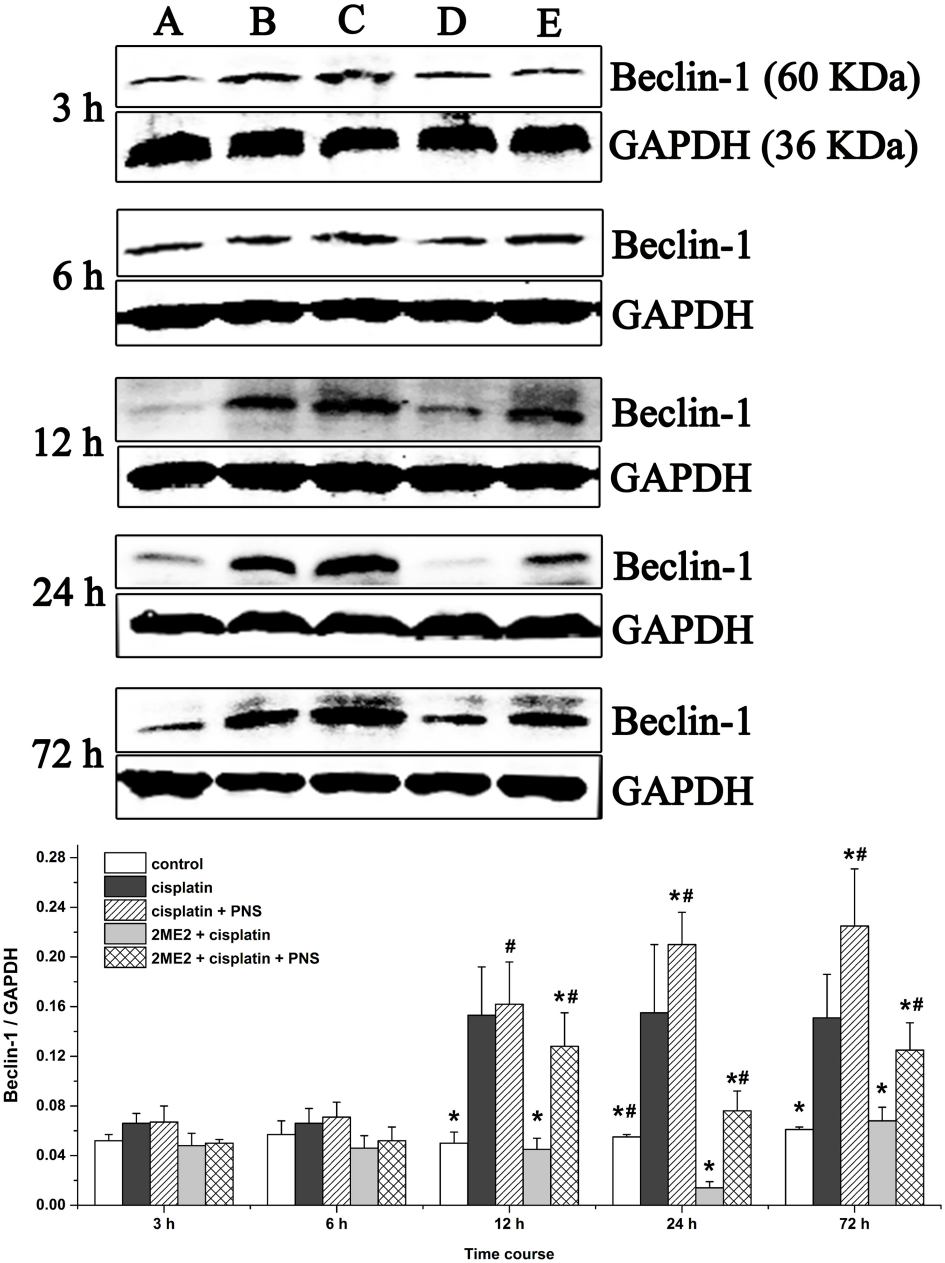
Western blotting analysis of Beclin-1 expression in rat renal tissues Beclin-1 expression in renal tissues from (**A)** control, (**B)** cisplatin, (**C)** cisplatin + PNS, (**D)** 2ME2 + cisplatin, (**E)** 2ME2 + cisplatin + PNS groups is shown. Data are represented as mean ± SD (n = 4). ^*^denotes *P* < 0.05 compared with the cisplatin group; ^#^ denotes *P* < 0.05 when compared with the 2ME2 + cisplatin group.

Western blotting analyses demonstrated increased Beclin-1 levels from 12h to 72h in the cisplatin group compared to the control (Figure 9). However, Beclin-1 levels were decreased in the 2ME2 + cisplatin group compared to the cisplatin group. Moreover, increased Beclin-1 levels were observed in the cisplatin + PNS group (24h and 72h) compared to the cisplatin group, as well as in the 2ME2 + cisplatin + PNS group (12h to 72h) compared to the 2ME2 + cisplatin group (Figure 9).

## DISCUSSION

The use of cisplatin, a widely used chemotherapeutic agent is limited due to its nephrotoxic effects. Cisplatin is largely taken up by renal tubular cells resulting in a dose-dependent decline in renal function [13]. In this study, we observed increased levels of Scr, BUN in blood and urinary NAG in cisplatin treated rats suggesting renal damage (Figure 1). These were also corroborated by high tubular injury scores based on analysis of H&E stained sections as well as TEM findings that demonstrated renal injury in cisplatin treated rats (Figure 2). These findings supported our previous findings [5] and suggested that cisplatin induced acute kidney injury (AKI). Furthermore, our results showed that HIF-1α inhibitor 2ME2 aggravated CIN, whereas PNS attenuated CIN (Figures 1 and 2), thereby suggesting the involvement of HIF-1α in alleviating CIN.

However, mechanisms of cisplatin-induced nephrotoxicity (CIN) have not yet been fully elucidated. Previous studies suggested that apoptosis [5], oxidative stress [3], DNA damage [14] and inflammation [4] were involved in CIN. In recent years, the role of autophagy in CIN has been recognized. Autophagy has been documented as renal response to cisplatin, protecting the kidneys from cisplatin injury [7, 15]. The conversion of LC3-I to LC3-II indicates autophagosome formation [16] and enhanced autophagy [17]. Additionally, TEM is the frequently used to monitor autophagosomes in addition to LC3 [18, 19]. We observed increased LC3-II and LC3-II/LC3-I ratio in the cisplatin group compared to the control group suggesting that autophagy was activated in response to cisplatin. We also showed that 2ME2 inhibited autophagy, whereas PNS enhanced autophagy (Figure 4). These results further corroborated TEM findings (Figure 3).

Autophagy is a mechanism for recycling cellular components [20]. It is activated in renal tubular cells in response to cisplatin. Enhanced autophagy can minimize cellular stress and accelerate recovery, thereby protecting renal function [21]. Furthermore, mitophagy maintains functional mitochondria by removing damaged mitochondrial proteins, DNA and membranes during CIN [22]. Inhibition of mitophagy leads to accumulation of damaged mitochondria and proximal tubule dysfunction [23]. Therefore, our results suggested that autophagy was activated to protect renal tissue from cisplatin injury. Additionally, 2ME2 significantly inhibits autophagy and aggravates the nephrotoxicity induced by cisplatin, thereby suggesting a role for HIF-1α in the recovery process. Also, PNS enhanced autophagy and reduced cisplatin-induced renal damage.

HIF-1α is a transcription factor that migrates to the nucleus and transcribes BNIP3 and other genes. Therefore, nuclear localization of HIF-1α and increased BNIP3 expression indicates activation of HIF-1α [24]. Cisplatin induces HIF-1α expression in renal tubular epithelial cells [25] as an adaptive response to hypoxia which is involved in the pathogenesis of CIN [26]. 2ME2 is an effective inhibitor of HIF-1α according to previous studies [27]. In our study, cisplatin increased the expression of HIF-1α mRNA (Figure 5) and protein (Figure 6) as well as the nuclear localization of HIF-1α (Figure 6) in the renal tissues. Moreover, HIF-1α mRNA and protein as well as the nuclear localization of HIF-1α was reduced by 2ME2, but increased by PNS. These results suggested that HIF-1α activity was involved in reducing the nephrotoxicity caused by cisplatin. In hypoxic conditions, HIF-1α migrates to the nucleus and transcribes target genes such as BNIP3 [28]. Therefore, increased BNIP3 expression indicates enhanced transcriptional activity of HIF-1α [29–31]. We observed increased BNIP3 mRNA (Figure 7) and protein (Figure 8) levels in the cisplatin treatment group. Conversely, BNIP3 mRNA and protein levels were decreased by 2ME2, but increased by PNS.

The HIF-1α/BNIP3 signaling pathway induces autophagy [32, 33]. BNIP3 is localized in the mitochondria and involved in autophagic clearance of the impaired mitochondria [34, 35]. Therefore, BNIP3 is a marker for evaluating mitophagy [36]. Beclin-1 is another key autophagy related protein. BNIP3 activates mitophagy by preventing the binding of Bcl-2 to Beclin-1, thereby freeing Beclin-1 to stimulate mitophagy [37–39]. Therefore, mitophagy requires HIF-1α-dependent expression of BNIP3 [31, 40] and Beclin-1 [31]. In our study, high Beclin-1 expression was observed in the cisplatin group (Figure 9). Further, Beclin-1 levels were decreased by 2ME2, but increased by PNS. The expression of Beclin-1, BNIP3 and HIF-1α concurred with the level of mitophagy activity in the different treatment groups at various time points. Therefore, our study demonstrated that PNS mitigated CIN by inducing mitophagy through the HIF-1α/BNIP3/Beclin-1 pathway.

## MATERIALS AND METHODS

### Animals

Male Sprague-Dawley (SD) rats weighing 200 ± 20g were obtained from the Experimental Animal Center of Guangxi Medical University (Guangxi, China). The animals were housed five per cage with a standard diet and water *ad libitum* and acclimatized for 1 week at 25 ± 5 °C and 60 ± 20% humidity. The animal experiments were conducted according to the protocols approved by the Animal Experimental Ethical Committee of Guangxi Medical University (approval No.: 201310009).

### Experimental design of drug administration

The rats were divided randomly into the following five groups: (1) control group, which received same volume of saline as cisplatin on day 1 and same volume of saline as PNS from days 1 to 3; (2) cisplatin group, which received a single dose of 5mg/kg cisplatin on day 1 and same volume of saline as PNS from days 1 to 3; (3) cisplatin + PNS group, which received a single dose of 5mg/kg cisplatin on day 1 and 31.35mg/kg PNS on days 1 to 3. (4) 2ME2 + cisplatin group which received a single dose of 4mg/kg 2ME2 half an hour before receiving a single dose of 5mg/kg cisplatin on day 1; they also received same volume of saline as PNS on days 1 to 3; (5) 2ME2 + cisplatin + PNS group, which received a single dose of 4mg/kg 2ME2 half an hour before receiving a single dose of 5mg/kg cisplatin on day 1; they also received 31.35mg/kg PNS on days 1 to 3.

PNS injection (batch number 14092108) was purchased from Wuzhou Pharmaceutical Co., Ltd. (Guangxi, China). 2-methoxyestradiol (2ME2, batch number 105M4158V) was purchased from Sigma-Aldrich Co. (St. Louis, MO). Cisplatin powder (batch number 5050272DB) was obtained from Qilu Pharmaceutical Co., Ltd. (Jinan, China). All drugs were administered through abdominal cavity injection. The cisplatin and PNS doses were according to our previous study [5, 12]. The dose of 2ME2 was according to previous reports [41, 42].

### Blood, urine and kidney specimen collection

Blood, urine and kidney samples were obtained at 3, 6, 12, 24 and 72h after treatments and stored at -80°C for analysis. After urine samples were collected, rats were sacrificed by injecting 30mg/kg sodium pentobarbital intraperitoneally. Then, the blood samples were obtained from abdominal aorta and centrifuged at 3000 rpm for 10 min at 4°C. The kidney tissue samples were washed with ice-cold saline; one portion was immersed immediately in 10% formalin solution for H&E staining and immunohistochemical detection, whereas the other portion was kept in 3% glutaraldehyde for transmission electron microscopy (TEM); the rest was stored immediately at -80°C for western blotting and for extracting RNA to detect HIF-1α mRNA and BNIP3 mRNA by qRT-PCR analysis.

### Analysis of BUN, Scr and urinary NAG levels

Serum creatinine (Scr), blood urea nitrogen (BUN) and urinary N-acetyl-β-D-glucosaminidase (NAG) levels were determined using the corresponding assay kits obtained from Nanjing Jiancheng Bioengineering Research Institute (Nanjing, China) according to manufacturer’s protocols. The Scr and BUN levels were measured in an automatic biochemical analyzer (7100, Hitachi, Japan).

### Hematoxylin and eosin (H&E) staining

Renal tissue samples were immersed in 10% formalin solution overnight at 4°C, and then embedded in paraffin and sectioned. Subsequently, the sections were stained with hematoxylin and eosin (H&E) and examined under a light microscope (IX51, Olympus, Japan). The renal tubular injury scores were determined according to previous studies [43]: score 0 = no damage; score 1 = mild (less than 10%); score 2 = moderate (10% to 25%); score 3 = severe (25% to 50%); score 4 = very severe (50% to 75%); score 5 = extensive damage (more than 75%). Ten randomly chosen fields (×400) were evaluated for each specimen and an average score was calculated. Histopathological changes were blindly scored by a pathologist.

### Transmission electron microscopy (TEM)

Renal tissue samples were fixed in 3% glutaraldehyde at 4°C for 2h and then paraffin embedded and sectioned. The samples were analyzed by transmission electron microscopy (Hitachi H7650, Japan) by an experienced pathologist who was blinded to this experiment.

### Western blotting analysis of LC3, BNIP3 and Beclin-1

Renal tissues samples from different sets of rats were homogenized using RIPA Lysis Buffer (50 mM Tris, 150 mM NaCl, 1% Triton X-100, 1% sodium deoxycholate, 0.1% SDS, and phosphatase and protease inhibitors) (Beyotime, Nanjing, China), and the protein lysates were quantified using BCA Protein Assay Kit (Beyotime, Nanjing, China) according to the manufacturer’s protocol. The protein lysates were separated on 12% SDS-PAGE (100 V for 2h) followed by transfer to PVDF membrane (Millipore, Billerica, MA) at 100 mA for 1h at 4°C. The PVDF membrane was then blocked by 5% skimmed milk for 1h followed by incubation with corresponding rabbit anti-rat primary antibodies overnight at 4°C. The antibodies included anti-LC3 (1:1000; 2775S, Cell Signaling, Danvers, MA), anti-BNIP3 (1:1000; 3769S, Cell Signaling, Danvers, MA) and anti-beclin-1 (1:1000; 3495S, Cell Signaling, Danvers, MA). Then, after washing, the membranes were incubated with the goat anti-rabbit fluorescent secondary antibody (1:15000; C30815-02, LICOR, USA) for 1.5h and analyzed by Odyssey infrared fluorescence scanning imaging system (Odyssey, LICOR, USA). GAPDH (1:10000; ab181602, Abcam, Cambridge, MA, USA) was used as internal control.

### Quantitative RT-PCR analysis

Renal tissues from the different rat groups were removed from -80°C to extract RNA. Total RNA was extracted using TRIzol reagent (Invitrogen, CA, USA) according to the manufacturer’s protocol. Total RNA (1μg) was reverse transcribed using Prime Script ™ RT reagent Kit with gDNA Eraser (Takara, Japan). Then, qRT-PCR was performed using a SYBR^®^ Premix Ex Taq ™ II kit (Takara, Japan) on an Applied Biosystems 7300 Real-Time PCR System (Applied Biosystems, MA, USA). The PCR primers are listed as follows: HIF-1α, 5’-TCTAGTGAACAGGATGGAATGGAG-3’(forward) and 5’-TCGTAACTGGTCAGCTGTGGTAA-3’(reverse); BNIP3,5’-TCTGGACGAAGCAGCTCCAA-3’(forward) and 5’- CCAAAGCTGTGGGTGTCTATTTCA-3’(reverse); GAPDH, 5’-GGCACAGTCAAGGCTGAGAATG-3’(forward) and 5’-ATGGTGGTGAAGACGCCAGTA-3’ (reverse). The PCR reaction mix included SYBR Premix Ex Taq II 10 ul, PCR Forward Primer (10 uM) 0.8 ul, PCR Reverse Primer (10 uM) 0.8 ul, ROX Reference Dye (50X) 0.4 ul, DNA 2 ul and RNase Free dH_2_O 6 ul. The PCR reaction was carried out as follows: 95°C for 30 s, 40 cycles of 95°C for 5 s, 60°C for 31 s. The expression of HIF-1α and BNIP3 relative to GAPDH was determined by 2^−ΔΔCt^ method, wherein ΔΔCt = (Ct _target_− Ct _GAPDH_) sample − (Ct _target_− Ct _GAPDH_) control.

### Immunohistochemical detection of HIF-1α

Paraffin-embedded kidney samples were sectioned, dewaxed and dehydrated. Then, the sections were incubated with 0.3% H_2_O_2_ to block the endogenous peroxidase activity and subsequently with 10% goat serum for 10 min at room temperature to block non-specific binding. After that, the sections were incubated with rabbit anti-rat HIF-1α primary antibody (1:1000; Cell Signaling, Danvers, MA) overnight at 4°C, and then incubated with biotinylated secondary antibody at 37°C for 1h and developed according to instructions using the DAB kit (Boster, Wuhan, China). After staining, tissue sections were visualized under a light microscope (Olympus Soft Imaging Solutions, Münster, Germany). The positively stained areas were quantified by measuring the integrated optical density (IOD) using Image Pro-plus 6.0 software. The nuclear staining of HIF-1α was also analyzed according to the previous reports [44]. Ten randomly chosen fields (×400) were evaluated for each specimen, and the average value was calculated. The protein expression of HIF-1α was presented as IOD/positive stained areas. The nuclear localization of HIF-1α was presented as the average number of positively stained nuclei.

### Statistical analysis

Statistical analysis was performed with the SPSS 19.0 software for Windows (SPSS Inc., Chicago, IL). The one-way analysis of variance (ANOVA) was used to analyze the differences between groups. The quantitative data were presented as the mean ± standard deviation (SD). The value of *P* < 0.05 was considered to be statistically significant.

### Supplementary information

For more information see Supplementary Materials.

## SUPPLEMENTARY MATERIALS FIGURES AND TABLE



## References

[1] Cleary JM, Mamon HJ, Szymonifka J, Bueno R, Choi N, Donahue DM, Fidias PM, Gaissert HA, Jaklitsch MT, Kulke MH, Lynch TP, Mentzer SJ, Meyerhardt JA (2016). Neoadjuvant irinotecan, cisplatin, and concurrent radiation therapy with celecoxib for patients with locally advanced esophageal cancer. BMC Cancer.

[2] Atilano-Roque A, Aleksunes LM, Joy MS (2016). Bardoxolone methyl modulates efflux transporter and detoxifying enzyme expression in cisplatin-induced kidney cell injury. Toxicol Lett.

[3] Zhu X, Jiang X, Li A, Zhao Z, Li S (2017). S-allylmercaptocysteine attenuates cisplatin-induced nephrotoxicity through suppression of apoptosis, oxidative stress, and inflammation. Nutrients.

[4] Humanes B, Camano S, Lara JM, Sabbisetti V, Gonzalez-Nicolas MA, Bonventre JV, Tejedor A, Lazaro A (2017). Cisplatin-induced renal inflammation is ameliorated by cilastatin nephroprotection. Nephrol Dial Transplant.

[5] Liu X, Huang Z, Zou X, Yang Y, Qiu Y, Wen Y (2014). Panax notoginseng saponins attenuates cisplatin-induced nephrotoxicity via inhibiting the mitochondrial pathway of apoptosis. Int J Clin Exp Pathol.

[6] Takahashi A, Kimura T, Takabatake Y, Namba T, Kaimori J, Kitamura H, Matsui I, Niimura F, Matsusaka T, Fujita N, Yoshimori T, Isaka Y, Rakugi H (2012). Autophagy guards against cisplatin-induced acute kidney injury. Am J Pathol.

[7] Hall AM, Schuh CD (2016). Mitochondria as therapeutic targets in acute kidney injury. Curr Opin Nephrol Hypertens.

[8] Shi X, Yu W, Yang T, Liu W, Zhao Y, Sun Y, Chai L, Gao Y, Dong B, Zhu L (2016). Panax notoginseng saponins provide neuroprotection by regulating NgR1/RhoA/ROCK2 pathway expression, *in vitro* and *in vivo*. J Ethnopharmacol.

[9] Yang Q, Wang P, Cui J, Wang W, Chen Y, Zhang T (2016). Panax notoginseng saponins attenuate lung cancer growth in part through modulating the level of Met/miR-222 axis. J Ethnopharmacol.

[10] Liu SJ, Zhou SW (2000). Panax notoginseng saponins attenuated cisplatin-induced nephrotoxicity. Acta Pharmacol Sin.

[11] Chen T, Li D, Fu YL, Hu W (2008). Screening of QHF formula for effective ingredients from Chinese herbs and its anti-hepatic cell cancer effect in combination with chemotherapy. Chin Med J (Engl).

[12] Liu X, Huang Z, Zou X, Yang Y, Qiu Y, Wen Y (2015). Possible mechanism of PNS protection against cisplatin-induced nephrotoxicity in rat models. Toxicol Mech Methods.

[13] Fahmi AN, Shehatou GS, Shebl AM, Salem HA (2016). Febuxostat exerts dose-dependent renoprotection in rats with cisplatin-induced acute renal injury. Naunyn Schmiedebergs Arch Pharmacol.

[14] Zhu S, Pabla N, Tang C, He L, Dong Z (2015). DNA damage response in cisplatin-induced nephrotoxicity. Arch Toxicol.

[15] Rovetta F, Stacchiotti A, Consiglio A, Cadei M, Grigolato PG, Lavazza A, Rezzani R, Aleo MF (2012). ER signaling regulation drives the switch between autophagy and apoptosis in NRK-52E cells exposed to cisplatin. Exp Cell Res.

[16] Maroni P, Bendinelli P, Resnati M, Matteucci E, Milan E, Desiderio MA (2016). The autophagic process occurs in human bone metastasis and implicates molecular mechanisms differently affected by Rab5a in the early and late stages. Int J Mol Sci.

[17] Wei F, Wang Y, Luo Z, Li Y, Duan Y (2017). New findings of silica nanoparticles induced ER autophagy in human colon cancer cell. Sci Rep.

[18] Martinet W, Schrijvers DM, Timmermans JP, Bult H, De Meyer GR (2013). Immunohistochemical analysis of macroautophagy: recommendations and limitations. Autophagy.

[19] Jin J, Zhan H, Lin B, Li Y, Zhang W, He Q (2017). Association of podocyte autophagosome numbers with idiopathic membranous nephropathy and secondary membranous nephropathy. Int Urol Nephrol.

[20] Xilouri M, Brekk OR, Polissidis A, Chrysanthou-Piterou M, Kloukina I, Stefanis L (2016). Impairment of chaperone-mediated autophagy induces dopaminergic neurodegeneration in rats. Autophagy.

[21] Yang Y, Song M, Liu Y, Liu H, Sun L, Peng Y, Liu F, Venkatachalam MA, Dong Z (2016). Renoprotective approaches and strategies in acute kidney injury. Pharmacol Ther.

[22] Stallons LJ, Funk JA, Schnellmann RG (2013). Mitochondrial homeostasis in acute organ failure. Curr Pathobiol Rep.

[23] Kimura T, Takabatake Y, Takahashi A, Kaimori JY, Matsui I, Namba T, Kitamura H, Niimura F, Matsusaka T, Soga T, Rakugi H, Isaka Y (2011). Autophagy protects the proximal tubule from degeneration and acute ischemic injury. J Am Soc Nephrol.

[24] Giatromanolaki A, Koukourakis MI, Sivridis E, Gatter KC, Harris AL, Banham AH (2006). Loss of expression and nuclear/cytoplasmic localization of the FOXP1 forkhead transcription factor are common events in early endometrial cancer: relationship with estrogen receptors and HIF-1alpha expression. Mod Pathol.

[25] Tanaka T, Kojima I, Ohse T, Inagi R, Miyata T, Ingelfinger JR, Fujita T, Nangaku M (2005). Hypoxia-inducible factor modulates tubular cell survival in cisplatin nephrotoxicity. Am J Physiol Renal Physiol.

[26] Eyvazzadeh N, Neshasteh-Riz A, Mahdavi SR, Mohsenifar A (2015). Genotoxic damage to glioblastoma cells treated with 6 MV X-Radiation in the presence or absence of methoxy estradiol, IUDR or topotecan. Cell J.

[27] Xin XY, Pan J, Wang XQ, Ma JF, Ding JQ, Yang GY, Chen SD (2011). 2-methoxyestradiol attenuates autophagy activation after global ischemia. Can J Neurol Sci.

[28] Klimova T, Chandel NS (2008). Mitochondrial complex III regulates hypoxic activation of HIF. Cell Death Differ.

[29] D'Hulst G, Ferri A, Naslain D, Bertrand L, Horman S, Francaux M, Bishop DJ, Deldicque L (2016). Fifteen days of 3,200 m simulated hypoxia marginally regulates markers for protein synthesis and degradation in human skeletal muscle. Hypoxia (Auckl).

[30] Wu H, Huang S, Chen Z, Liu W, Zhou X, Zhang D (2015). Hypoxia-induced autophagy contributes to the invasion of salivary adenoid cystic carcinoma through the HIF-1alpha/BNIP3 signaling pathway. Mol Med Rep.

[31] Zhang H, Bosch-Marce M, Shimoda LA, Tan YS, Baek JH, Wesley JB, Gonzalez FJ, Semenza GL (2008). Mitochondrial autophagy is an HIF-1-dependent adaptive metabolic response to hypoxia. J Biol Chem.

[32] Ishihara M, Urushido M, Hamada K, Matsumoto T, Shimamura Y, Ogata K, Inoue K, Taniguchi Y, Horino T, Fujieda M, Fujimoto S, Terada Y (2013). Sestrin-2 and BNIP3 regulate autophagy and mitophagy in renal tubular cells in acute kidney injury. Am J Physiol Renal Physiol.

[33] Schonenberger MJ, Kovacs WJ (2015). Hypoxia signaling pathways: modulators of oxygen-related organelles. Front Cell Dev Biol.

[34] Chinnadurai G, Vijayalingam S, Gibson SB (2008). BNIP3 subfamily BH3-only proteins: mitochondrial stress sensors in normal and pathological functions. Oncogene.

[35] Wei H, Liu L, Chen Q (2015). Selective removal of mitochondria via mitophagy: distinct pathways for different mitochondrial stresses. Biochim Biophys Acta.

[36] Cui J, Shi S, Sun X, Cai G, Cui S, Hong Q, Chen X, Bai XY (2013). Mitochondrial autophagy involving renal injury and aging is modulated by caloric intake in aged rat kidneys. PLoS One.

[37] Eng CH, Abraham RT (2011). The autophagy conundrum in cancer: influence of tumorigenic metabolic reprogramming. Oncogene.

[38] Lin YF, Chiu IJ, Cheng FY, Lee YH, Wang YJ, Hsu YH, Chiu HW (2016). The role of hypoxia-inducible factor-1alpha in zinc oxide nanoparticle-induced nephrotoxicity *in vitro* and *in vivo*. Part Fibre Toxicol.

[39] Zhang J, Ney PA (2009). Role of BNIP3 and NIX in cell death, autophagy, and mitophagy. Cell Death Differ.

[40] Shelby SJ, Angadi PS, Zheng QD, Yao J, Jia L, Zacks DN (2015). Hypoxia inducible factor 1alpha contributes to regulation of autophagy in retinal detachment. Exp Eye Res.

[41] Yeh CH, Chou W, Chu CC, So EC, Chang HC, Wang JJ, Hsing CH (2011). Anticancer agent 2-methoxyestradiol improves survival in septic mice by reducing the production of cytokines and nitric oxide. Shock.

[42] Zou X, Zhou L, Zhu W, Mao Y, Chen L (2016). Effectiveness of 2-methoxyestradiol in alleviating angiogenesis induced by intracranial venous hypertension. J Neurosurg.

[43] Kim J, Long KE, Tang K, Padanilam BJ (2012). Poly(ADP-ribose) polymerase 1 activation is required for cisplatin nephrotoxicity. Kidney Int.

[44] Kairaitis LK, Wang Y, Gassmann M, Tay YC, Harris DC (2005). HIF-1alpha expression follows microvascular loss in advanced murine adriamycin nephrosis. Am J Physiol Renal Physiol.

