# Supplementary material for: Panax notoginseng saponins mitigate cisplatin induced nephrotoxicity by inducing mitophagy via HIF-1α
Source: Oncotarget. 2017 Aug 3;8(61):102989–3003. doi: 10.18632/oncotarget.19900 (PMC5732705; doi:10.18632/oncotarget.19900)
Supplement: Supplementary file 1 [file oncotarget-08-102989-s001.pdf]

Panax notoginseng saponins mitigate cisplatin induced nephrotoxicity by inducing mitophagy via HIF-1α

SUPPLEMENTARY MATERIALS

PNS increases the lysosomes in cisplatin treated renal tubular epithelial cells

The experimental design is the same as that in the Manuscript.

TEM images (Supplementary Figure 1) revealed that lysosomes increased in the cisplatin group (24 h and 72 h), the cisplatin + PNS group (12 h to 72 h) and the 2ME2 + cisplatin + PNS group (24 h and 72 h).

Effects of PNS on oxidative stress in rat renal tissues

The experimental design is the same as that in the Manuscript, including the grouping of animals, the drug treatments, the collections of specimens, and so on.

Kidney samples were obtained at 3, 6, 12, 24 and 72h after treatments. After kidney sample homogenized, the liquid supernatant was collected and detected for oxidative stress indexes, including reactive oxygen species (ROS), malondialdehyde (MDA) and superoxide dismutase (SOD) using the assay kits. All performed procedures were strictly in accordance with the kit requirements.

The cisplatin group showed higher ROS (6h to 72h) and MDA (24h and 72h) levels compared to the control group. Compared with the cisplatin group, the 2ME2 + cisplatin group rats showed further elevated ROS and MDA levels at 24h and 72h compared to the cisplatin group. However, the cisplatin + PNS group demonstrated lower ROS and MDA levels at 72h compared to the cisplatin group. The 2ME2 + cisplatin + PNS group also demonstrated lower ROS and MDA levels at 24h and 72h compared to the 2ME2 + cisplatin group (Supplementary Figure 2A and Supplementary Figure 2B).

Besides, the cisplatin group showed lower SOD level at 24h and 72h compared to controls. Compared with the cisplatin group, the 2ME2 + cisplatin group rats showed further decreased SOD level at 24h and 72h. However, the cisplatin + PNS group demonstrated higher SOD levels at 72h compared to the cisplatin group. The 2ME2 + cisplatin + PNS group also demonstrated higher SOD levels at 72h compared to the 2ME2 + cisplatin group (Supplementary Figure 2C).

Effects of PNS on the levels of VEGF mRNA and VEGF protein

The experimental design is the same as that in the Manuscript, including the grouping of animals, the drug treatments, the collections of specimens, and so on.

Kidney samples were obtained at 3, 6, 12, 24 and 72h after treatments. The VEGF mRNA and VEGF protein levels were detected by qRT-PCR and western blot, respectively.

The PCR primers are listed as follows: VEGF, 5’- TCCTGCAGCATAGCAGATGTGA -3’ (forward primer) and 5’- CCAGGATTTAAACCGGGATTTC -3’ (reverse primer); GAPDH, 5’-GGCACAGTCAAG GCTGAGAATG-3’ (forward primer) and 5’-ATGGTGG TGAAGACGCCAGTA-3’ (reverse primer). The expression of VEGF relative to GAPDH was determined by $2^{-\Delta\Delta Ct}$ method, wherein $\Delta\Delta Ct = (Ct_{target} - Ct_{GAPDH})$ sample $- (Ct_{target} - Ct_{GAPDH})$ control.

VEGF is one of the downstream targets of HIF-1α. From 12 h to 72 h, the VEGF mRNA level was higher in the cisplatin group compared to the control group, while lower in the 2ME2 + cisplatin group compared to the cisplatin group (Supplementary Figure 3). Similarly, at 24 h and 72 h, the VEGF protein level was higher in the cisplatin group compared to the control group, while lower in the 2ME2 + cisplatin group compared to the cisplatin group (Supplementary Figure 4). Conversely, VEGF mRNA and VEGF protein levels were higher in the cisplatin + PNS group (24 h and 72 h) compared to the cisplatin group, as well as in the 2ME2 + cisplatin + PNS group (24 h and 72 h) compared to the 2ME2 + cisplatin group (Supplementary Figure 3 and Supplementary Figure 4).

2ME2 has not itself toxicity on rat kidney

The rats were divided randomly into the control group and the 2ME2 group in our early stage work. The rats in the control group received the same volume of saline as 2ME2 on day 1; the rats in the 2ME2 group received a single dose of 4mg/kg 2ME2 on day 1. All drugs were administered through abdominal cavity injection.

Blood, urine and kidney samples were obtained at 72h after treatments. Next, Serum creatinine (Scr), blood urea nitrogen (BUN) and urinary N-acetyl-β-D-glucosaminidase (NAG) levels, as well as kidney hematoxylin and eosin (H&E) staining were detected.

As shown in the Supplementary Table 1 and Supplementary Figure 5, Scr, BUN and urinary NAG levels as well as the tubular injury scores were not significant difference in the 2ME2 group when compared with the control group (Supplementary Table 1, Supplementary Figure 5).

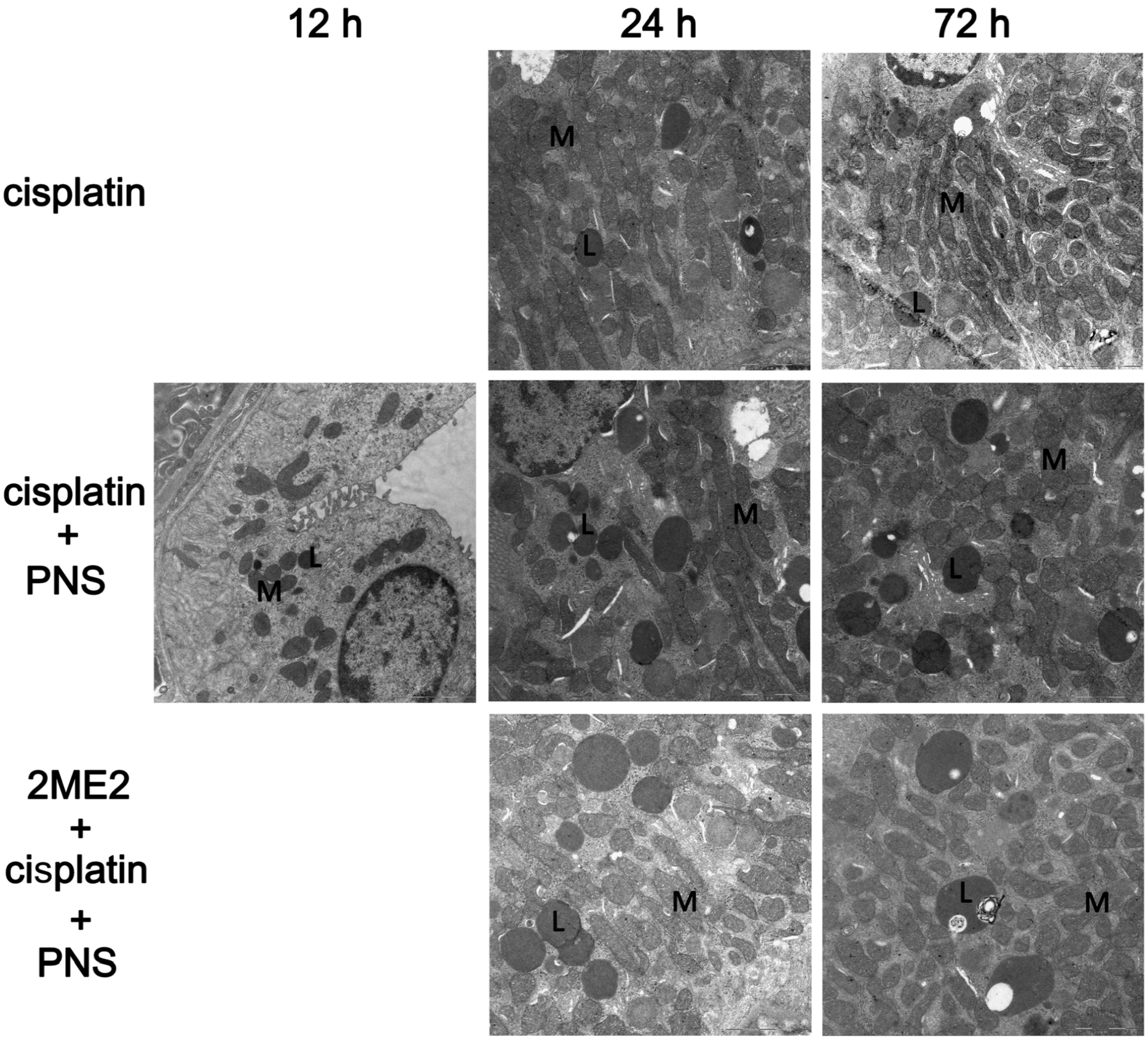

Supplementary Figure 1: Transmission electron micrographs of rat renal tissue sections (20000×). Representative TEM images of renal tissue sections from cisplatin, cisplatin + PNS and 2ME2 + cisplatin + PNS groups of rats at different time points are shown (scale:1 μm). Note: M denotes mitochondria; L denotes lysosomes.

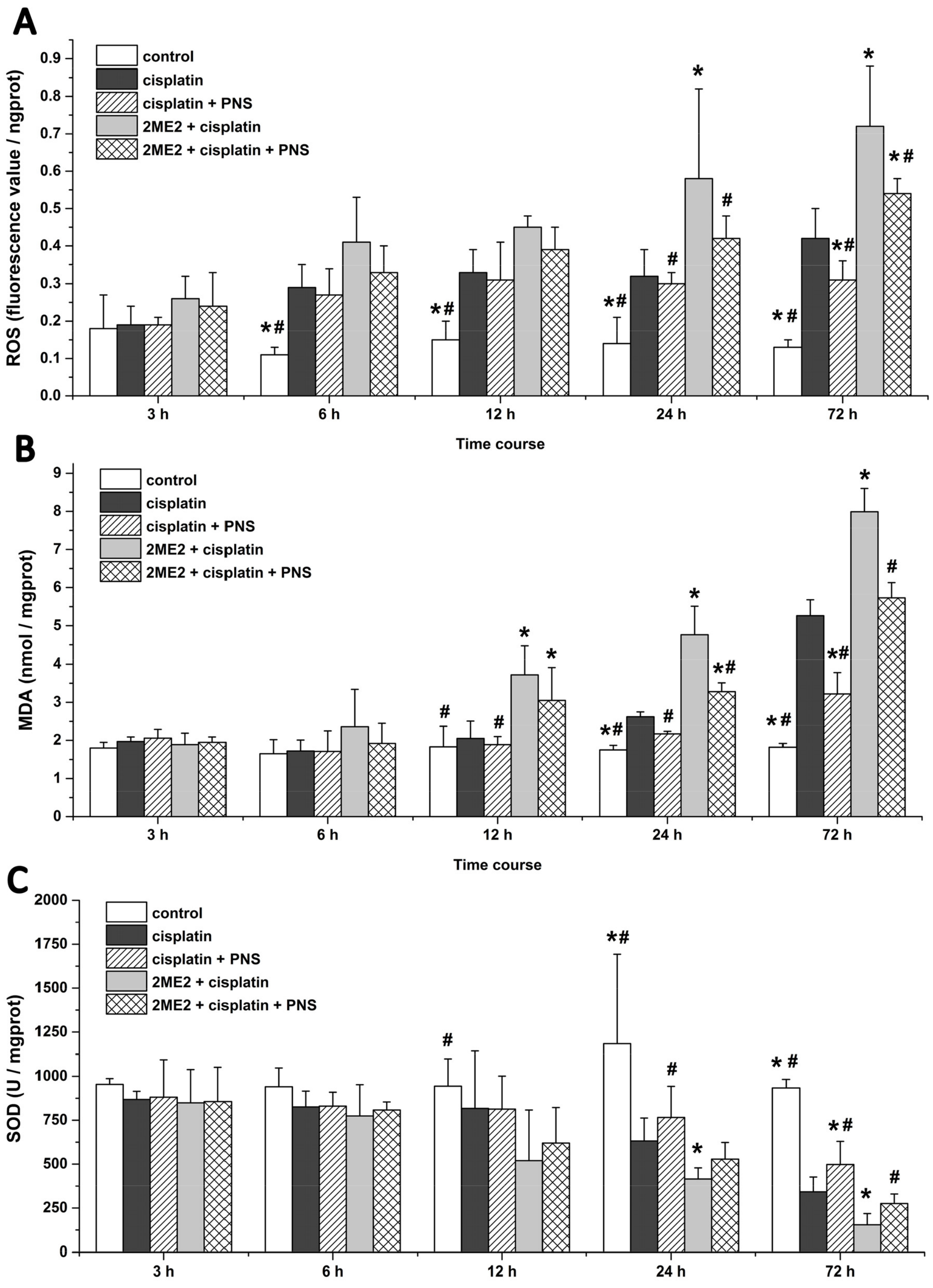

Supplementary Figure 2: ROS, MDA and SOD levels in renal tissue at different time points. **(A)** ROS, **(B)** MDA, **(C)** SOD levels. Note: Data are represented as mean ± SD (n = 6). * denotes $P < 0.05$ compared with the cisplatin group, # denotes $P < 0.05$ compared with the 2ME2 + cisplatin group.

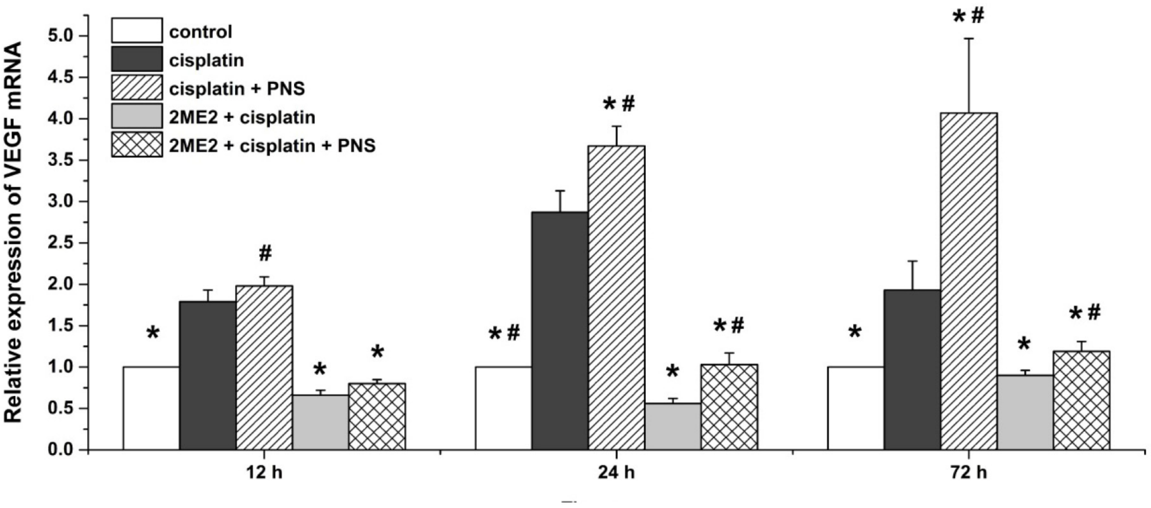

Supplementary Figure 3: The VEGF mRNA expression in rat renal tissue. Note: Data are represented as mean ± SD (n = 3). * denotes $P < 0.05$ compared with the cisplatin group; # denotes $P < 0.05$ when compared with the 2ME2 + cisplatin group.

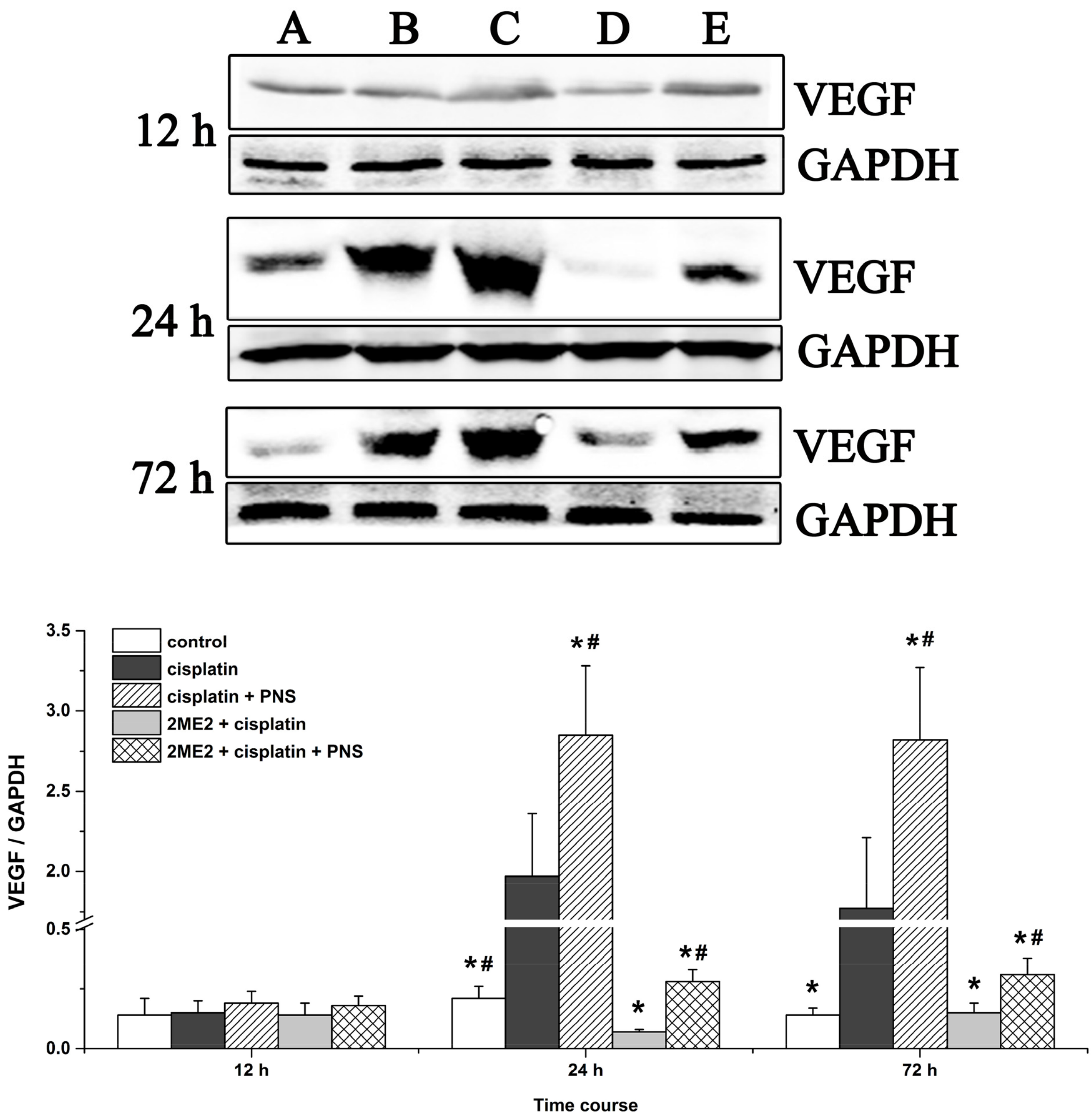

Supplementary Figure 4: Western blot analysis of VEGF expression in rat renal tissues. Quantitative analysis of VEGF expression in renal tissues from **(A)** control, **(B)** cisplatin, **(C)** cisplatin + PNS, **(D)** 2ME2 + cisplatin, **(E)** 2ME2 + cisplatin + PNS groups is shown. Note: Data are represented as mean ± SD (n = 3). * denotes $P < 0.05$ compared with the cisplatin group; # denotes $P < 0.05$ when compared with the 2ME2 + cisplatin group.

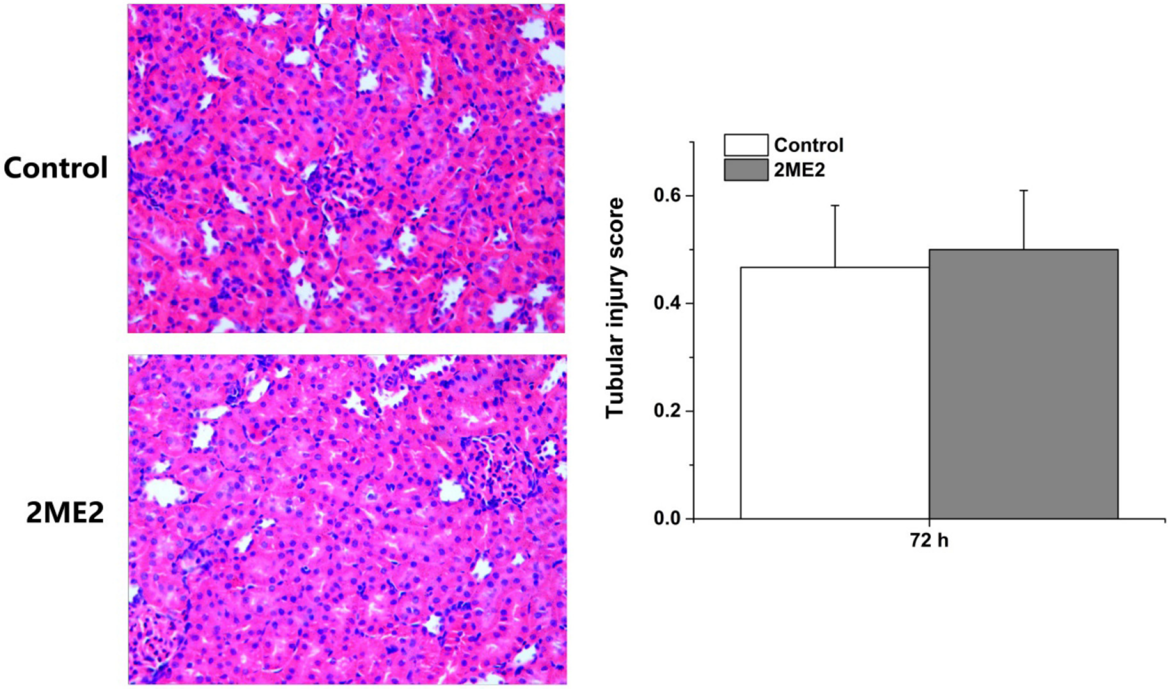

Supplementary Figure 5: H&E stained renal tissue samples and their tubular injury scores. The structures of kidney tissues and the tubular injury scores in the 2ME2 group were no abnormal change or not significantly changed when compared with the control group, respectively. Note: Data are represented as mean ± SD (n = 6).

Supplementary Table 1: The levels of Scr, BUN and urinary NAG

	Scr (umol / L)	BUN (mmol / L)	NAG (U / L)
The control group	38.50 ± 4.42	4.41 ± 1.61	1.97 ± 0.62
The 2ME2 group	37.83 ± 3.66	4.28 ± 0.99	1.99 ± 0.41

Note: Data are represented as mean ± SD (n = 6).
